# Synthesis of Cobalt–Nickel Aluminate Spinels Using the Laser-Induced Thermionic Vacuum Arc Method and Thermal Annealing Processes

**DOI:** 10.3390/nano12213895

**Published:** 2022-11-04

**Authors:** Rodica Vladoiu, Aurelia Mandes, Virginia Dinca, Elena Matei, Silviu Polosan

**Affiliations:** 1 Faculty of Applied Sciences and Engineering, Dept of Physics, Ovidius University of Constanta, Mamaia Av. No 124, 900527 Constanta, Romania; 2 Academy of Romanian Scientists, Splaiul Independentei Street No. 54, 050094 Bucharest, Romania; 3 National Institute of Materials Physics, P.O. Box MG-7, 077125 Bucharest-Magurele, Romania

**Keywords:** cobalt–nickel aluminate, Laser-induced Thermionic Vacuum Arc, thermal annealing, cermet

## Abstract

To obtain highly homogeneous cobalt–nickel aluminate spinels with small crystallite sizes, CoNiAl alloy thin films were primarily deposited using Laser-induced Thermionic Vacuum Arc (LTVA) as a versatile method for performing processing of multiple materials, such as alloy/composite thin films, at a nanometric scale. Following thermal annealing in air, the CoNiAl metallic thin films were transformed into ceramic oxidic (Co,Ni)Al_2_O_4_ with controlled composition and crystallinity suitable for thermal stability and chemical resistance devices. Structural analysis revealed the formation of (Co,Ni)Al_2_O_4_ from the amorphous CoNiAl alloys. The mean crystallite size of the spinels was around 15 nm. Thermal annealing induces a densification process, increasing the film thickness together with the migration process of the aluminum toward the surface of the samples. The sheet resistance changed drastically from 200–240 Ω/sq to more than 10^6^ Ω/sq, revealing a step-by-step conversion of the metallic character of the thin film to a dielectric oxidic structure. These cermet materials can be used as inert anodes for the solid oxide fuel cells (SOFCs), which require not only high stability with respect to oxidizing gases such as oxygen, but also good electrical conductivity. These combination metal–ceramics are known as bi-layer anodes. By controlling the crystallite size and the interplay between the oxide/metal composite, a balance between stability and electrical conductivity can be achieved.

## 1. Introduction

Rapid advancements in the field of batteries in the electrical automotive industry require efficient solutions with respect to aspects such as energy storage efficiency, constructive characteristics, cost price, safety and usage life [1]. Lithium-ion batteries have become the most widely used technology in electric vehicles, thanks to both their high energy density and their increased power per mass battery unit, allowing the development of batteries with reduced weight and dimensions at competitive prices. These batteries are based on a liquid electrolyte, making them extremely heavy and dangerous. A common trend is the direction of attention toward high-temperature solid oxide fuel cell (SOFC) batteries, which represent the most efficient and versatile electrochemical energy conversion system [2]. These solid oxide fuel cells contain a standard anode, a cathode, and a solid-state oxide electrolyte. While the most common electrolyte is based on yttria-stabilized zirconia (YSZ), different anode and cathode materials have been proposed to increase the number of charging and discharging cycles. For anodes, Ni-based cermets are the most common materials, because of the reducing conditions of the fuel gas [3,4].

Cermets consist of a ceramic matrix bonded by a metallic binder. The metal improves the toughness and thermal shock resistance of the ceramic [5]. Metals are used as the matrix phase, and the reinforcement phase is comprised of ceramic.

Among cermets, Ni- NiAl_2_O_4_ has been proposed as an anode for SOFC systems, because NiAl_2_O_4_ presents a degree of inversion and its efficiency depends mainly on the physical union of triple phase boundaries and a porosity of around 40% [6,7,8]. This is because Ni-based materials have excellent electro-catalytic activity for the purposes of the H_2_ oxidation reaction, high electrical conductivity, and high stability with YSZ electrolyte [9,10].

Nickel aluminate spinels are currently used due to their high thermal stability and melting point, mechanical stability, and resistance to alkalis and acids [11]. Based on their defect chemistry, these spinels are widely used in catalytic applications and, along with Li ions in NiAl_2_O_4_, are candidates for the development high-temperature fuel cells [12]. These applications are correlated with the spinel structure, which presents high surface area, thermal stability and chemical resistance [13].

The replacement of the nickel ions with cobalt ions substantially modifies the material properties of the anode, because cobalt is another suitable SOFC anode material, since the metal is able to withstand the fuel environment while remaining non-oxidized [14]. Moreover, cobalt has the advantage of high sulfur tolerance, and the oxidation potential of cobalt is higher than that of nickel, thus requiring less complete fuel combustion.

Several techniques can be used for the production of nickel aluminate spinel, with the most common being the combustion method [15], chemical co-precipitation [16,17], microwave heating from a coprecipitated mixture of nickel and aluminum hydroxides precursor [18], the sol–gel auto combustion method and calcination [19], or the simple sol–gel method and calcination [20]. All of these methods result in pure or doped NiAl_2_O_4_ ceramic materials, and to a lesser extent, the Ni-NiAl_2_O_4_ cermet materials, which are very useful for anode technologies.

CoNiAl metallic films were obtained using the Laser-induced Thermionic Vacuum Arc (LTVA), which is based on the anodic arc discharge and an electron gun system. When a high DC voltage is applied to the anode, the electrons coming from the electron gun are accelerated and ionize the vapors above the anode, in order to ignite the plasma. In LTVA technology, the atoms of the base material are moved into the plasma column via photonic processing due to the adjustable power laser beam, replacing a portion of the original atoms (to be deposited) to ionize and conduct electricity.

Following thermal annealing in air, the CoNiAl metallic thin films were transformed into ceramic oxidic (Co,Ni)Al_2_O_4_ with controlled composition and crystallinity suitable for thermal stability and chemical resistance devices and anode technologies. A structural analysis of the metallic thin films and the subsequent cermet structures obtained by thermal annealing was performed using XRD, SEM, EDX techniques combined with electrical and dielectric measurements.

## 2. Materials and Methods

In order to obtain a compact cermet structure and a highly homogeneous cobalt–nickel aluminate spinel with small crystallite sizes, we propose a new technique, Laser-induced Thermionic Vacuum Arc (LTVA), by which the CoNiAl alloy thin films are primarily deposited [21,22]. In addition to the typical system described by the term TVA [23,24,25,26], in the LTVA configuration, the laser beam is provided by a QUANTEL Q-Smart 850 Nd:YAG compact Q-switched laser with a second harmonic module. The principle of the TVA is based on the combination of the anodic arc with a powerful electron gun system. It is a versatile method for performing multimaterial processing, such as in the case of alloy/composite thin films at a nanometric scale [27,28,29,30,31].

The main experimental parameters used in this study are summarized in Table 1. The cathode acts as an electron gun heated by the intensity current of the filament and the anode is a carbon crucible, filled with a mixture of Co granules (99.95% metal basis), Ni granules (99.996%, metal basis), and aluminum granules (99.999%, metal basis) in a weight ratio of 1:1:1. The experimental setup for binary/ternary mixtures has been described elsewhere [32,33,34]. After ignition of the plasma, the power applied on the arc discharge was P = 375 W, where P = U_a_·I_a_; the symbols U_a_ and I_a_ represent the arc voltage and the arc current during deposition, respectively.

CoNiAl alloy thin films were deposited on the Si/SiO_2_ (100) substrates, Prime CZ-Si wafer, with thickness of 275 ± 25 μm, (100), polished on one side, p-type (Boron) doped and with a, SiO_2_ thickness of 500 nm. The wafer was cut into 15 × 15 mm^2^ pieces and cleaned using the standard method (deionized water and alcohol) before deposition. The CoNiAl alloy thin film thickness was monitored using a Cressington Thickness Monitor quartz balance during deposition, and the estimated thickness was 145 nm.

**Figure 1:**
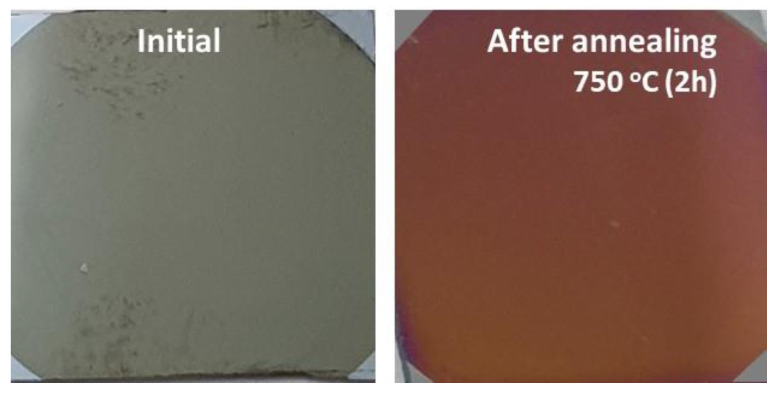
Photos of the CoNiAl samples before and after annealing.

The annealing procedure was performed in a Nabertherm 1300 furnace in air with a heating rate of 6.5 °C/min, up to 750 °C, and maintained at this temperature for 2 h, before being slowly cooled at the same heating rate to room temperature. Figure 1 shows the photos of the CoNiAl sample before and after the annealing procedure.

X-ray diffraction patterns were obtained using a Bruker D8 Advance diffractometer with CuK_α1_ radiation (λ = 1.54056 Å) at 40 kV and 40 mA. The analysis of the obtained peaks was performed after subtraction of the Kα1 lines from the Kα1-Kα2 doublet, using the Bruker Difracplus Basic Evaluation program. The 2θ scan range was set at 15°–60°, with a step size of 0.05° and a resolution of 0.01°.

The morphologies of the samples were studied using a Carl Zeiss EVO 50XVP scanning electron microscope (SEM) and a field emission scanning electron microscope (FESEM) Gemini 500, Carl Zeiss.

Direct Current (DC) electrical measurements were performed using an Ossila four-point probe with distances of 1.27 mm between the probes, which had a round tip of 0.24 mm, giving the probes a larger contact surface area than a needle would, thereby spreading out the downward force being applied to the sample. The voltage applied on the outer probes was between 0 and 10 V. Each measurement was repeated ten times to obtain a mean value.

Dielectric measurements were performed at room temperature with a UC2878 precision LCR meter, at frequencies between 20 Hz and 1 MHz. The real and imaginary parts of the impedance were extracted from the capacitance–frequency (C-f) curves, based on the geometry of the sample (15 × 15 mm^2^ and thicknesses extracted from SEM images). The fitting procedure was performed using Eisanalyzer software.

## 3. Results

### 3.1. Structural Analysis (XRD; SEM; EDX)

**Figure 2:**
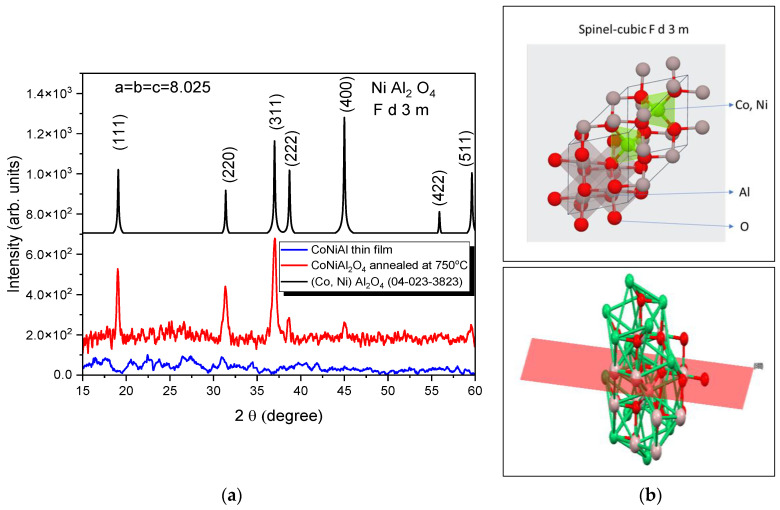
X-ray patterns of initial and annealed films (**a**) and the obtained spinel structure (**b**). Interstitial positions partially occupied by tetrahedral- Co or Ni and octahedral-Al atom (**up**). Distinguishing the normal spinels is the perpendicular growth of the (400) plane from 45.04° as seen in the XRD results (**down**).

The structural properties of the CoNiAl alloy thin film and the subsequent thermally annealed sample were characterized by XRD (Figure 2a), revealing the amorphous character of the alloy thin film, while the annealed sample exhibited a partially crystalline structure, as evidenced by the diffraction peaks centered at (311). The experimental patterns were fitted with Gaussian functions to extract the peak position, half-width, intensity, and spacing layers.

The effective crystallite size of the annealed sample was calculated based on the line broadening of the XRD peaks of the spinel structure (Figure 2b). In a single line analysis, the apparent crystallite size D is related to the Gaussian diffraction peak at the Bragg angle θ, following the Debye–Scherrer equation. The linear dimension of (Co, Ni) Al_2_O_4_ based on the (311) reflection peak is D = 15 nm.

The fitting procedure, based on the first five peaks, reveals a cubic structure with the following parameters: a = b = c = 8.025 Å and α = β = γ = 90°. The crystallization fraction, given by the ratio between the areas under the XRD peaks of crystalline cubic structures and the total area under the XRD curve, is in the range around 18% for two-hour annealing at 750 °C.

### 3.2. Surface Elemental Analysis (SEM/EDX)

**Figure 3:**
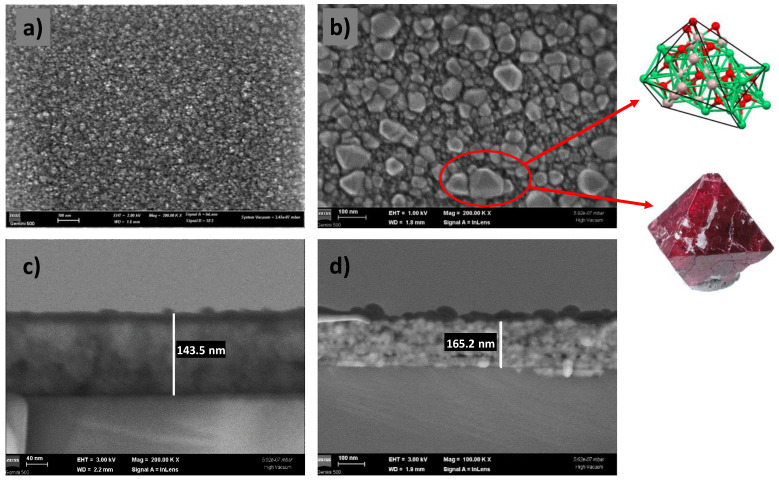
SEM images of the CoNiAl samples, before (**a**) and after (**b**) annealing, with cross-section details (**c**,**d**).

The bright-field and dark-field images of the CoNiAl thin-film alloy were recorded at a low operating voltage of 2 kV, allowing a better surface analysis, and showed an amorphous but irregular surface. The optical gray surface of the CoNiAl thin-film alloy exhibits a non-uniform surface with a certain roughness (around 4–5 nm) (Figure 3a).

In the annealed samples, the surface becomes more structured, showing some regular geometries. The operating voltage used was the same, at 2 kV, but in order to obtain a better view of the crystallite edges, the voltage was then lowered to 1 kV, keeping the magnification and the working distance at the same values of 200.00 KX Mag and 1.8 mm. The nanocrystal sizes vary between 50 and 100 nm, with some of them presenting a bipyramidal structure (Figure 3b).

Moreover, the annealing procedure is accompanied by a densification of the film, and the initial thickness of around 145 nm increases to 160–165 nm, as revealed by the cross-section SEM images (Figure 3c,d).

**Figure 4:**
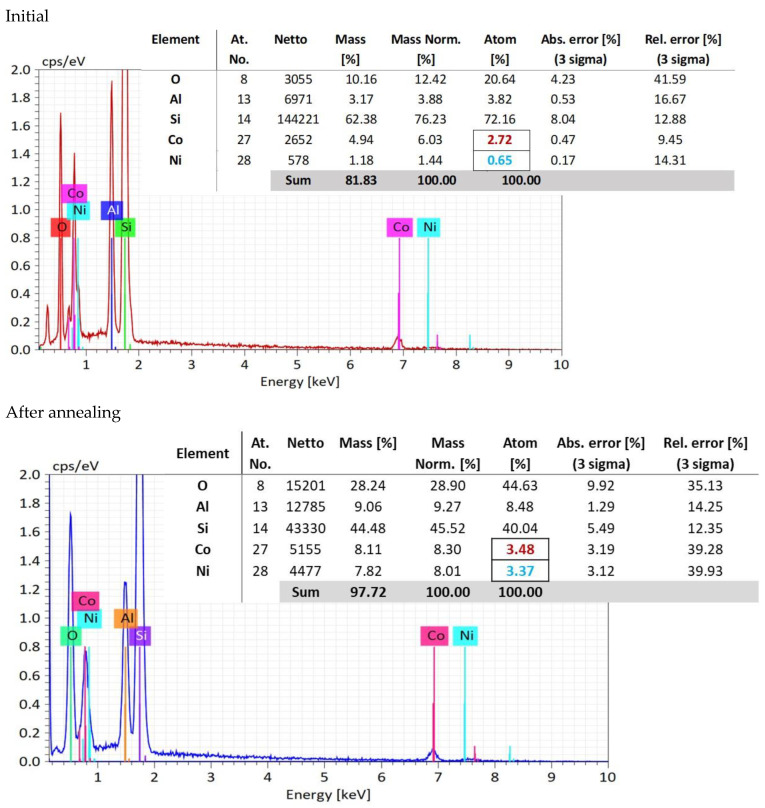
EDX analysis measured at three points, and compositional analysis.

In the EDX measurements (Figure 4), it is shown that in the oxygen atomic concentration increases from a mean value of 21.66 atomic percent to 44.63 atomic percent, because of the thermal oxidation, indicating the oxygen pick up from air during annealing.

Meanwhile, the concentration of aluminum ions, as a mean value of the three selected areas, increases from 3.82 to 8.48. This fact indicates a migration process of the aluminum atoms toward the sample surface, favoring the conversion of the CoNiAl alloy into (Co, Ni) Al_2_O_4_, which requires a higher aluminum and oxygen content.

Concerning the ratio between Co:Ni atomic percentage distribution, the initial atomic percentage in CoNiAl alloy is 4:1.

However, after annealing in air, oxygen increases from an atomic percentage of 28.81 to 44.63, indicating the oxygen pick up from air during annealing. After thermal annealing, the atomic percentage concentrations for Co and Ni are almost the same, suggesting an equal contribution to the crystallography of (Co, Ni) Al_2_O_4_ cermet substrate. The initial atomic percentage in CoNiAl alloy is 3:1.

### 3.3. Electrical Measurements

**Figure 5:**
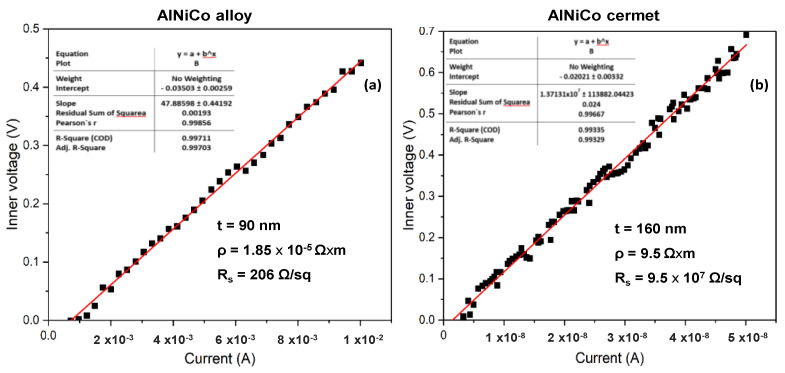
Sheet resistance of (**a**) the initial CoNiAl and (**b**) the annealed sample.

The four-point probe measurements on the initial CoNiAl thin-film alloy suggests a slight increase in the sheet resistance, around 200–240 Ω/sq, depending on the area of the measurements, because the sheet resistance is higher in the center of the sample due to the non-uniform alloying distribution of the atoms (Figure 5a). This fact leads to a thin film resistivity between 1.8 and 2.16 ∗ 10^−5^ Ω ∗ m, based on the following relation:

(1) $$\rho =R_{s}*t\;[Ω*m]$$

where *t*-is the thickness of the sample, which is around 145 nm, and *R_s_* is the sheet resistance, which is given by:

(2) $$R_{s}=\frac{\pi }{\ln2}*\frac{\Delta V}{\Delta I}*C\;[Ω/\mathrm{sq}]\;$$

The above equation accounts for the slope between the inner voltage and the applied current on the inner electrodes of the four-point probe equipment. Here, *C* is a correction factor for a square sample of 15 × 15 mm^2^, with a value of 0.95.

For the annealed sample, based on the same measurement parameters, the sheet resistance drastically increases up to 5.9 – 6 × 10^7^ Ω/sq, marking the transformation of the metallic film into a dielectric structure with much lower electrical conductivity (Figure 5b).

### 3.4. Dielectric Measurements

**Figure 6:**
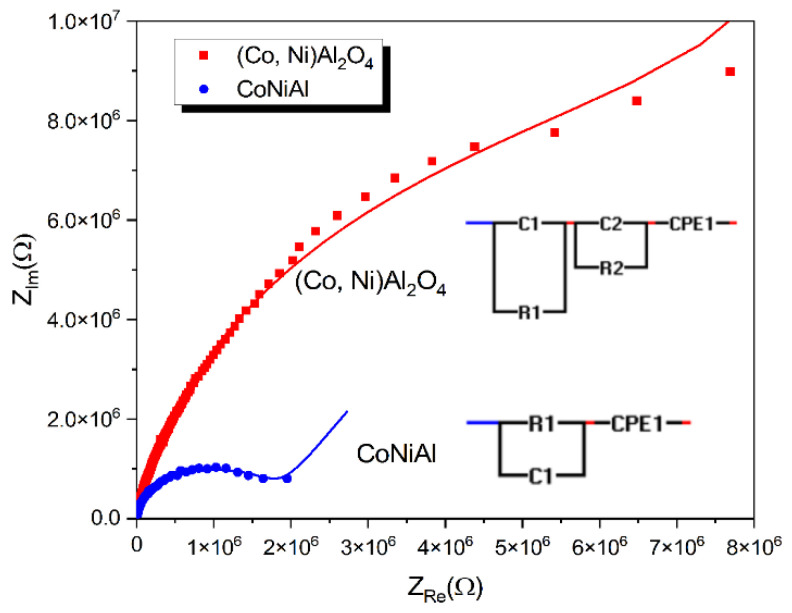
Complex dielectric behaviors before and after annealing.

Dielectric measurements were performed using a metal–oxide–semiconductor (MOS) structure, with a p-type silicon substrate doped with boron and 500 nm SiO_2_ as the dielectric substrate. The initial thickness of 145 nm of the CoNiAl thin-film alloy was used as the metallic electrode. After the thermal annealing procedure, the resulting partially crystalline (Co, Ni) Al_2_O_4_ cermet adds dielectric properties to the MOS structure. Figure 6 presents the complex impedances for the initial CoNiAl thin-film alloy and the annealed sample, while the fitting parameters are given in Table 2.

For the initial metal alloy, the electric circuit describes the role of the SiO_2_ dielectric layer given by the (R_1_, C_1_) parallel circuit, which remain the same before and after annealing. The Constant Phase Element (*CPE*) defines the nonideal capacitances that may be caused by inhomogeneities in the surface of metal–oxide electrodes, resulting in a nonideal capacitance in the double-layer at the solid/electrolyte interface. The impedance of a *CPE* is defined by the following relation [35,36]:

(3) $$Z_{CPE}=\frac{1}{{\left(j\omega \right)}^{\beta }Q}$$

where *Q* is the nonideal capacitance, and has units of F s*^β^*^−1^, and *β* is an ideality factor that ranges from 0 to 1. For *β* = 0, the element is purely resistive, while *β* = 1 represents an ideal capacitor.

As can be seen, the resistance of the annealed sample increases to 6 MΩ and the high frequency arc in this modulus plot is mainly caused by the grain boundary capacitance, which is modeled by introducing the R_2_-C_2_ parallel circuit.

## 4. Discussion

The XRD patterns of the CoNiAl alloy thin film show an amorphous structure, meaning that the LTVA deposition method in a vacuum does not lead to the direct crystallization of the deposited layers. The post-annealing crystallography of the deposited films revealed an Fd-3m space group structure similar to that of NiAl_2_O_4_, with the origin at 3. This structure is characteristic of the direct spinels, in which the occupancies of the Ni^2+^ and Al^3+^ cations follow tetrahedral and octahedral sites (see Figure 2b), assuming a degree of inversion around 0.8. This degree of inversion is related to the general formula of these oxides, which is as follows:

(A_1−x_B_x_)[4][A_x_B_2−x_][6]O_4_

where A is given by Ni^2+^ and B by the Al^3+^. The normal spinel is defined for x = 0, with the formula NiAl_2_O_4_, while the inverse spinel is obtained for x = 1 with the formula Al [NiAl]O_4_.

The cationic substitution of the Ni^2+^ with Co^2+^ does not significantly change the crystallographic structure of the spinel, because the atomic radii of the Co and Ni cations are the same, at around 1.35 Å [37]. However, the lattice parameter of the (Co, Ni) Al_2_O_4_ is lower than that of the NiAl_2_O_4_ spinel cubic structure, with a lattice parameter varying between 8.046 and 8.051 Å. This is probably due to the incomplete formation of the cubic structure for a quite fast annealing time at 750 °C (2 h) compared with the prolonged time of NiAl_2_O_4_, which is around 320 h at 800 °C. The average crystallite size is ~15 nm, which is very similar to those obtained by mechanical milling for 40 h and subsequent annealing at 600 °C of (Co,Ni)Al_2_O_4_ spinel powders, starting with the complex intermetallic compound Al_70_Co_15_Ni_15_ [38].

The crystallographic parameters are similar to those obtained by Roelofsen et al. for the NiAl_2_O_4_, which were determined on the basis of equimolar amounts of NiO and Al_2_O_3_ preheated in air at 800–1000 °C for 24 h, followed by annealing from 800 to 1500 °C [39]. Rietveld analysis of the XRD patterns for the powder annealed between 800 and 1500 °C revelaed a cubic structure for the NiAl_2_O_4_ and a lattice parameter that varies from 8.046 Å at 800 °C to 8.051 Å at 1500 °C. It is clear that the substitution of Ni ions with Co ions slightly reduces the cell parameters to 8.025 Å.

The thermally annealed CoNiAl thin-film alloy leads to a partial crystalline structure (Co, Ni) Al_2_O_4_, very similar to those of NiAl_2_O_4_ and MgAl_2_O_4_ direct spinels, in which the estimated crystalline fraction is in the range around 18%. The annealing procedure results in an increase in the film thickness from 145 nm to around 160 nm. The surface of the annealed thin film shows the formation of nanocrystallites with dimensions of up to 100 nm. The crystallography of these crystallites is very similar to that of MgAl_2_O_4_ direct spinels as a twin boundary (Figure 3, inset). The twinning process is governed by the spinel-twin law, which leads to face-centered cubic (FCC) minerals [40].

The thermal annealing, even for a short time, changed the color of the deposited thin film from a gray color characteristic of metallic thin films to the brown-red color of (Co, Ni) Al_2_O_4_ thin films, which is comparable to the color of MgAl_2_O_4_ natural spinel (Figure 1). The mapping process confirmed the uniform distributions of the Ni and Co, and the process of diffusion of the Al atoms toward the surface of the sample during the annealing process, thus facilitating the formation of the spinel structure.

More interesting is the evolution of the atomic concentrations before and after thermal annealing. While in the CoNiAl thin-film alloy the ratio between the Co and Ni atomic concentrations is around 4:1, suggesting a composition of Co_0.8_Ni_0.2_Al, in the thermally annealed sample, this ratio is almost 1:1 (3.48% Co, 3.37% Ni), leading to a (Co_0.5_Al_0.5_) Al_2_O_4_ structure (marked with colors in Figure 4). This fact suggests the rearrangement of the Co and Ni atoms to fulfill the stoichiometry of the (Co, Ni) Al_2_O_4_, leaving probably a large quantity of cobalt atoms in their metallic form. Meanwhile, the increase in the aluminum content on the surface of the sample is evidenced by its having the same stoichiometry as the (Co, Ni) Al_2_O_4_, and could be observed on the basis of the cross-section EDX mapping measurements. This is beneficial for the inert anodes in which the lithium atoms can be replaced with more stable aluminum atoms.

The electrical resistivity is substantially higher in the initial thin films compared with the bulk metals due to their having different mechanisms of electron scattering. This resistivity is dependent on the thickness of the metallic film, which should be compared with the electron mean free path, accounting for electron scattering with interfaces and defects [41]. For a bulk metal, it is necessary to assume that the thickness of the mean free path of the electrons is on the order of several hundred interatomic distances under ordinary temperatures, and increases rapidly in pure metals reach reaching very low temperatures. Considering the normal propagation of the current on the metallic thin-film surface for a standard classical four-point probe measurement, the main contribution of the current is given by the electrons, which move in parallel with the metal surface, rather than with the bulk one [42].

The resistivity of the annealed sample is around 9.5 to 10 Ω*m, which is lower than that of ceramics, but much higher than that of the metallic thin films, by six orders of magnitude. The electrical conductivity is somewhere around 0.012 S/cm, which is two orders of magnitude below the electrical conductivity of 3.36 S/cm obtained for the NiFe_2_O_4_ thin films [43].

In the MOS configuration, the SiO_2_ dielectric layer given by the (R1, C1) parallel circuit remains the same in both the CoNiAl thin-film alloy and the partial crystalline (Co, Ni) Al_2_O_4_ cermet, in which the metal contribution acts as the electrode. However, a small contribution from the possible oxidation of the metallic surface cannot be excluded, and probably occurs as a result of the oxidation of the metals in the initial sample.

In both cases, the surface roughness and heterogeneities are marked by nonideal capacitances, which may be caused by the inhomogeneity and porosity of the deposited alloy and interfaces formed during the crystallization process.

In the thermally annealed sample, a new dielectric contribution is formed when the partial crystalline (Co, Ni) Al_2_O_4_ cermet is formed. This fact is modeled using a new (R_2_, C_2_) parallel circuit which is characterized by its higher resistive contribution and a capacitive contribution in the range of a few pF. The resistive contribution is similar to that measured by the DC electrical measurements, showing the formation of a dielectric aluminate crystallin structure. The capacitive contribution of the aluminate crystallin structure is somehow similar to the case of pure oxide ceramics like Y_2_O_3_-doped ZrO_2_, which are widely used as nanocrystalline thin-film electrolytes [44]. This capacitance is attributed to the grain boundary contribution of the small (Co, Ni) Al_2_O_4_ crystallites.

Concerning exponent β for the constant phase element, a gradual increase from 0.7 to 0.9can be observed, indicating the transformation of the structure from a more resistive to a capacitive one due to the formation of the aluminate crystallin structure. For ceramics, β is found to be in the range 0.8−1, indicating nonideal behavior that is attributed to surface roughness and irregularities in surface termination, porosity, and complexity in the double-layer structure [35]. However, for the zirconia (YSZ) layers, this exponent varies between 0.7 to 0.8 for the grain boundary capacitances [45].

## 5. Conclusions

The method of metallic deposition of alloys using LTVA technology, followed by the gradual transformation into a dielectric aluminate crystalline structure, represents an alternative method for obtaining thin-film electrolytes or even anodes.

The gradual thermal annealing, in time for the CoNiAl thin-film alloy to transform the sample into a cermet (Co, Ni)Al_2_O_4_ structure, even within a short period of time and at a relatively lower temperature, was demonstrated on the basis of the X-ray patterns, in which around 18% of the alloys exhibited the cubic structure of spinels. The SEM images confirmed the formation of a bipyramidal structure, while the EDX analysis confirmed the formation of (Co, Ni) Al_2_O_4_, which is similar to the natural MgAl_2_O_4_ spinel.

The thermal annealing of the metallic CoNiAl thin-film alloy changed the electrical behaviors of the initial metallic alloy, which had lower resistivity, into a ceramic structure with a resistivity of up to 10^7^ Ω/sq. Dielectric measurements confirmed the formation of a second oxidic layer on the basis of the presence of a new capacitive contribution, similar to that shown by previous ceramic materials.

In this way, the electro-catalytic activity for the H_2_ oxidation reaction, high electrical conductivity, and high stability of the Ni-based materials are combined with the non-oxidizing, high sulfur tolerance and the oxidation potential of cobalt-based materials, increasing the anode capability of the obtained cermet materials.

## Figures and Tables

**Table 1:** List of the experimental parameters for depositing CoNiAl thin films.

Parameters	
Energy/pulse of laser E (mJ)	100
Irradiance of the laser I_L_ (GW/cm^2^)	20
Base pressure p_B_ (Pa)	5 × 10^−6^
Working pressure p_W_ (Pa)	4.7 × 10^−5^
Intensity current on the filament I_F_ (A)	58
Film thickness t (nm)	145
Time of deposition (s)	900

**Table 2:** Complex impedance analysis.

Parameter	CoNiAl Alloy	(Co, Ni)Al_2_O_4_ Cermet
**C_1_**	2.4 pF	2.7 pF
**R_1_**	1.82 MΩ	2.2 MΩ
**C_2_**	-	7.3 pF
**R_2_**	-	8.06 MΩ
**CPE**	980 pF	16.8 pF
**β**	0.7	0.9
